# Early loss of vascular‐astrocyte contact in the hippocampus in Alzheimer’s disease. Data from patients, in vitro and in vivo experimental models

**DOI:** 10.1002/alz.092730

**Published:** 2025-01-03

**Authors:** Flavia Eugenia Saravia

**Affiliations:** ^1^ University of Buenos Aires, Buenos Aires Argentina

## Abstract

**Background:**

Alzheimer’s disease is characterized by the accumulation of aggregated amyloid peptides in the brain parenchyma and in the walls of brain vessels. The hippocampus ‐ a complex brain structure that plays a key role in learning and memory ‐ has been implicated in the disease. However, there is limited data on vascular changes during the pathological degeneration of Alzheimer’s disease in this vulnerable structure, which has distinctive vascular features.

**Method:**

Our aim was to evaluate vascular changes in the hippocampus of AD patients and PDAPP‐J20 mice ‐ a model of AD ‐ and to determine the impact of Aβ40 and Aβ42 on endothelial cell activation.

**Result:**

We found a loss of physical astrocyte‐endothelium interaction in the hippocampus of individuals with AD compared to non‐AD donors, along with reduced vessel density. Astrocyte‐endothelial interactions and levels of the tight junction protein occludin were altered early in PDAPP‐J20 mice, preceding any signs of morphological changes or blood‐brain barrier dysfunction in these mice. At later stages, PDAPP‐J20 mice showed reduced hippocampal vascular density and leakage of fluorescent tracers, indicating vascular and BBB dysfunction. In vitro studies showed that exposure to soluble Aβ40 in human brain microvascular endothelial cells (HBMEC) was sufficient to induce NFκB translocation to the nucleus, which may be related to the observed reduction in occludin levels. Inhibition of the membrane receptor for advanced glycation endproducts (RAGE) prevented these changes in HBMEC. Additional results suggest that Aβ42 indirectly affects the endothelium by inducing astrocytic factors.

**Conclusion:**

Our results from human and mouse brain samples provide evidence for the critical involvement of the hippocampal vasculature in Alzheimer’s disease.

